# Exploring and analysing single cell multi-omics data with VDJView

**DOI:** 10.1186/s12920-020-0696-z

**Published:** 2020-02-18

**Authors:** Jerome Samir, Simone Rizzetto, Money Gupta, Fabio Luciani

**Affiliations:** 1 School of Medical Sciences and Kirby Institute for Infection and Immunity, UNSW Sydney, Sydney, Australia; 2 Garvan Institute of Medical Research, Sydney, Australia

**Keywords:** Immune cells, scRNA-seq, T cell receptor, B cell receptor, Multi-omics

## Abstract

**Background:**

Single cell RNA sequencing provides unprecedented opportunity to simultaneously explore the transcriptomic and immune receptor diversity of T and B cells. However, there are limited tools available that simultaneously analyse large multi-omics datasets integrated with metadata such as patient and clinical information.

**Results:**

We developed VDJView, which permits the simultaneous or independent analysis and visualisation of gene expression, immune receptors, and clinical metadata of both T and B cells. This tool is implemented as an easy-to-use R shiny web-application, which integrates numerous gene expression and TCR analysis tools, and accepts data from plate-based sorted or high-throughput single cell platforms. We utilised VDJView to analyse several 10X scRNA-seq datasets, including a recent dataset of 150,000 CD8^+^ T cells with available gene expression, TCR sequences, quantification of 15 surface proteins, and 44 antigen specificities (across viruses, cancer, and self-antigens). We performed quality control, filtering of tetramer non-specific cells, clustering, random sampling and hypothesis testing to discover antigen specific gene signatures which were associated with immune cell differentiation states and clonal expansion across the pathogen specific T cells. We also analysed 563 single cells (plate-based sorted) obtained from 11 subjects, revealing clonally expanded T and B cells across primary cancer tissues and metastatic lymph-node. These immune cells clustered with distinct gene signatures according to the breast cancer molecular subtype. VDJView has been tested in lab meetings and peer-to-peer discussions, showing effective data generation and discussion without the need to consult bioinformaticians.

**Conclusions:**

VDJView enables researchers without profound bioinformatics skills to analyse immune scRNA-seq data, integrating and visualising this with clonality and metadata profiles, thus accelerating the process of hypothesis testing, data interpretation and discovery of cellular heterogeneity. VDJView is freely available at https://bitbucket.org/kirbyvisp/vdjview.

## Background

Immunological studies have revealed a surprisingly high level of heterogeneity between immune cells, even in those with same clonotype and surface phenotype, suggesting that lymphocyte populations of apparently similar phenotype could have different functions [1]. With the advent of single cell RNA-sequencing (scRNA-seq), it is now possible to unravel the heterogeneity of T and B cells and link receptor clonotype diversity to the gene expression profile of each cell and to clinical or other metadata. Multi-modality single cell datasets are rapidly pervading in medical research, and are being used to identify novel cellular states and molecular features of diseases [2–4], to extract information on the DNA (mutations, methylation), mRNA (gene expression profiles) and to further study the heterogeneity of immune cells of apparently similar clonotype and phenotype [3].

With the recent availability of scRNA-seq derived clonal and transcriptomic data, several software packages have been developed for the downstream analyses of these data types [3]. For instance software packages such as TRACER [5] BRACER [4] and VDJPuzzle (for both TCR [6] and BCR [2]) can accurately identify the full-length TCR and BCR from the sequenced cDNA. A vast set of tools are already available to perform gene expression analysis, including clustering, differential expression, dimensionality reduction, trajectory inference, and gene signature identification (e.g. https://www.scrna-tools.org/). More recently, epitope barcoding on cell surface has been also integrated with scRNA-seq, further highlighting the importance of multi-modal single cell technologies [7, 8].

Integrating these levels of genomic information can be important to fully decipher the changes of immune cells during immune response, or to identify subsets of rare cells with specific phenotypes. Tools that integrate several of the available methods to analyse single cell transcriptomics have been proposed [9, 10]. Additionally, it is often necessary to link this information with clinical and other metadata, for instance with the tissue origin, surface phenotype (e.g. flow cytometry data at the time of index sorting), or with the sample origin and disease diagnosed. To date, there are limited software packages that are accessible to non-bioinformatics experts, and that allow simultaneous analysis of gene expression, immune receptors and notably clinical and other metadata. For instance, Loupe Cell Browser 3.1 from 10X Genomics provides users with a first line of analysis to explore gene expression and annotate their dimensionality reduction plots with immune receptor information. However, such tools do not permit extensive analysis of the data, such as hypothesis testing and integration of metadata into differential expression or immune receptor analyses. Additionally, such tools usually have strict input requirements, with Loupe Cell Browser 3.1 not allowing users to analyse datasets from different technologies, such as plate-based sorting, which remains a common technology of choice to study immune repertoires.

Multi-layer analyses often require lengthy integration of bioinformatics and biological skills. Experience with software tools, such as R packages, is often a barrier to entry, with most of the data manipulation, visualisation and package integration being left to the user. To properly answer and address biological questions, multiple packages need to be complemented with ad hoc scripts that modify input data, filter cells and then test hypotheses, which is a source of latency between the biologist and the bioinformatician. Here, we report VDJView, a shiny app that delivers an integrated set of novel and publicly available tools to analyse and visualise the clonal and transcriptomic data with clinical and metadata. VDJView addresses the drawbacks in currently available multi-omics analysis tools, by removing the need for a skilled bioinformaticians and allowing researchers to test hypotheses and explore the relationship between multi-modal single cell datasets.

## Implementation

**Fig. 1:**
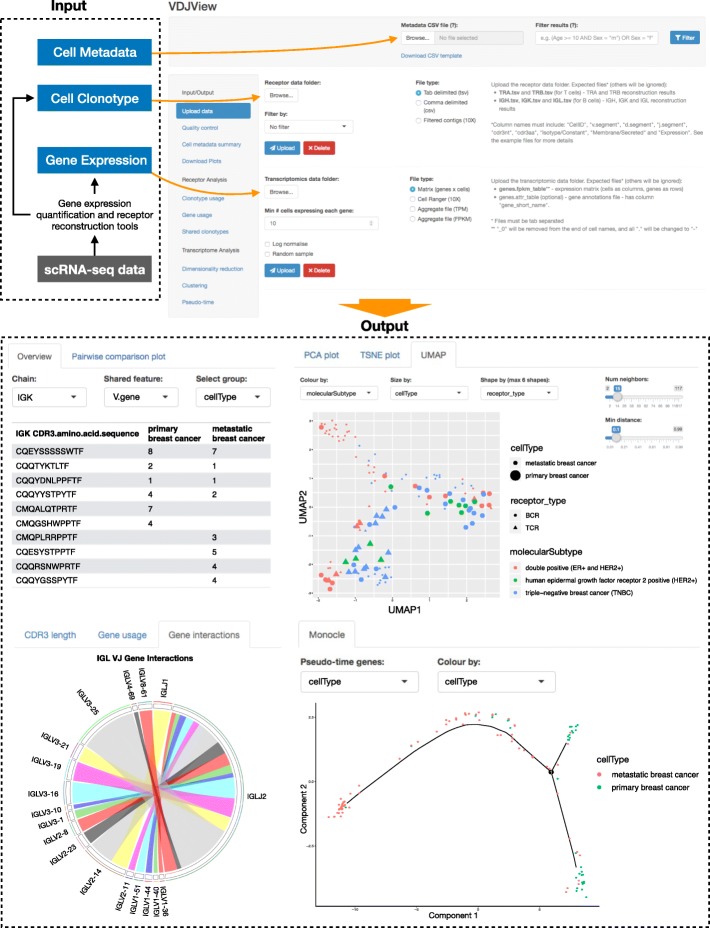
Overview of VDJView. Top: VDJView upload page, showing where required (immune receptor sequences and gene expression matrix) and optional inputs (metadata) can be uploaded. Bottom: examples of analysis using scRNA-seq from primary cancer tissues and metastatic lymph-node revealing clonally expanded T and B cells. The table (top left) shows a clonal expansion of IGL chains across primary breast tissue and metastatic lymph-node. The Circos plot (bottom left) shows the IgL V and J gene pairings identified. Dimensionality reduction using UMAP (top right) shows a cluster of B cells derived from metastatic lymph-node in two patients with ER^+^ HER2^+^ breast cancer, while T and B cells from the primary breast cancer tissue had similar gene signature regardless of molecular subtype. Pseudo-time plot (bottom right) shows the inferred evolutionary trajectory between all immune cells determined by genes that differentiate primary from metastatic tissues in two subjects with matched samples

VDJView is an R Shiny web-application developed for the analysis of clonal and transcriptomic single-cell data (Fig. 1). The intuitive graphical user interface allows researchers with or without computational training to interactively analyse and explore their datasets, interrogating the results against user uploaded cell metadata. VDJView acts as a wrapper for commonly used transcriptomic and receptor analysis packages (Table 1), integrating them and allowing the user to generate and manipulate figures and tables. The plots generated are exportable to publication-quality pdf files, and all tables can be downloaded in csv format.

**Table 1:** List of modules implemented in VDJView with their outputs and integrated packages

Module	Description	Software packages	Output
Filtering	Selection of cells based on metadata, gene and immune receptor features	dplyr	Venn Diagram, data-table
Quality control	Metrics with options for easily filtering cells according to total read counts, number of genes, and percentage of mitochondrial/ribosomal genes	Seurat [11]	Violin plots
Random sampling	Selection of small subsets of data, providing the ability to analyse larger datasets	Seurat	
Clonotype usage	Pie charts of single- and paired-chain CDR3 contig usage for both T and B cells. Tables detailing single- and paired-chain CDR3 contigs generated across all cells	plotly	Pie charts, data-tables
CDR3 length	Distribution of CDR3 lengths for single- and paired chains	tcR	Histograms
VDJ gene usage	Distribution of V, D and J gene usage for single chains	tcR	Histograms
Gene interactions	Frequencies of inter- and intra-chain VDJ gene pairings, and inter-chain CDR3 pairings	Rcircos [12]	Circos plots
Shared clonotypes	Table and scatter plot detailing the number of single- and paired-chain CDR3 contigs and VDJ genes that occur in multiple subgroups, and their frequency in each group	tcR	Scatter plot, data-table
Dimensionality reduction	PCA plot, t-SNE plot and UMAP plot with customisable parameters. Metadata can be used to control data point shape, size and colour. Data points are selectable and displayed with their metadata in a data-table below each plot	Scater [13], Seurat, SC3 [14]	PCA plots, t-SNE plots, UMAP plots, data-tables
Unsupervised clustering	Consensus matrix, gene expression heatmaps and marker-gene heatmaps are calculated by SC3 based on user defined cluster ranges, p-values and AUROC values. Metadata can be displayed above plot. Gene list can be uploaded to generate an expression heatmap. Tabular SC3 clustering information is generated	Scater, SC3	Consensus matrix, Expression matrix, DE Genes heatmap, Marker genes heatmap, data-table
Supervised clustering	Differentially expressed gene heatmap generated by MAST comparing groups of cells based on clusters predetermined by the user, p-value and fold change thresholds. Gene fold change values and a tabular version of the heatmap are generated	MAST [15], Scater, pheatmap	Gene expression matrix, data-tables
Pseudo-time	Pseudo-time plot to determine single-cell state trajectories based on genes which are differentially expressed between user defined metadata groups	Monocle [16]	Pseudo-time cell trajectory plot
Cell metadata summary	Tabular summary of the cells uploaded, the metadata associated with them and the number of receptors contigs, and expressed genes reported for each cell		Data-table

VDJView has been extensively tested on Linux and MacOS, with most features functional on Windows as well, and has the sole requirement of an R version of at least 3.5.2 being installed. VDJView has been tested on multiple datasets available from published literature using SmartSeq2 and 10X libraries (see below). On a machine with 32GB RAM, a dataset of 5000 cells takes 1 min to upload, and most plots render instantaneously with the exception the PCA (principle component analysis), TSNE (t-distributed stochastic neighbour embedding) and UMAP (uniform manifold approximation and projection) plots which take about 20 s to render. The clustering and pseudo-time plots can take 20–25 min to calculate. Larger datasets have been uploaded, however, with transcriptomic data on over 50,000 genes for more than 20,000 cells, 32GB of RAM is insufficient.

### VDJView input data

Pre-analysed scRNA-seq data can be directly uploaded into VDJView. The three data types that VDJView accepts are; T and/or B cell receptor data, gene expression data, and metadata. Immune receptor data can be uploaded as a list in csv or other tabular formats. Gene expression data can be uploaded as a matrix of expression counts per cell or other common formats including those generated by the 10X Cell Ranger kit. Metadata can be uploaded in csv format. Cells can be filtered according to their metadata and the presence of a TCR/BCR, meaning that multiple analyses can be performed without needing to re-upload a dataset. An example of this is when the user uploads data from multiple subjects, VDJView allows cells from individual subjects of interest to be filtered in/out. VDJView can also be pipelined with computational tools that generate gene expression and immune receptor sequencing from raw data, thus permitting user-defined workflow. Here, we have tested VDJView with scRNA-seq data available publicly and generated by high-throughput 3′ or 5′ end technologies, 10X and SmartSeq2 data.

### Datasets analysed

1. SmartSeq2 breast cancer T and B cells, N = ~ 560 [17]
2. 10X CD8+ T cells, N = ~ 150,000 (https://www.10xgenomics.com/resources/application-notes/a-new-way-of-exploring-immunity-linking-highly-multiplexed-antigen-recognition-to-immune-repertoire-and-phenotype/). The entire TCR datasets of donors 1 and 2 were analysed. For gene expression analysis, a random sample of 15,000 cells for each of donors 1, 2 and 3 were considered.

### VDJView features and modules

VDJView integrates multiple R software packages to provide a powerful yet cohesive repertoire of analysis modules (Table 1). Numerous interactive and customizable figures are provided for the analysis of clonotype data, and further modules are available for the simultaneous or isolated exploration of expression data. All figures and tables are updated automatically if any of the relevant parameters are changed during the analysis. Further details and a complete list of features can be found in Supplementary Note 1.

## Results

### Analysis of SmartSeq2 breast cancer cells

To demonstrate the utility and novelty of VDJView, we analysed scRNA-seq data (full-length transcriptome, SmartSeq2 protocol) from the primary breast tissues and metastatic lymph nodes of 11 subjects [17]. We input the original, unfiltered scRNA-seq data (*N* = 563 cells) into VDJPuzzle [2] to quantify the gene expression and reconstruct the TCR and BCR, parsing the results into VDJView. We found 170 single B cells with at least one full-length H, L or K chain, of which 101 had a full-length heavy and light chain. Similarly, we found 42 single T cells with at least one full-length α or β TCR chain, of which 30 had paired TRα and TRβ chains. Thus, we have uniquely identified T and B cells via their receptor, confirming the findings of the authors of the original work who identified T and B cells through gene enrichment analysis [17]. In addition to these, we found 33 cells with TCR and BCR chains, suggesting that they were likely contaminants or doublets. Of the 34 single cells filtered out in the original publication due to sequencing quality, VDJPuzzle reconstructed a BCR for two cells, and partially reconstructed the BCR in 12 others. While our analysis of the T cells revealed a highly diverse repertoire (Supplementary Figure 1), we identified a clone in BC03 which was present in both primary and metastatic lymph node tissues, as well as 31 B-cell clones, with clonotypes shared across primary and metastatic tissues, and across subjects (Fig. 1 and Supplementary Figures 1 and 2, Supplementary Tables 1 and 2). This type of analysis was not performed in the original publication [17] and further demonstrates the utility of VDJView.

To further complement the work done by Chung et al. [17], we performed dimensionality reduction (Supplementary Figure 3) and a pseudo-time analysis on these immune cells, showing that a common repertoire of B cells is involved in breast cancer with a migratory pattern between primary and metastatic tissues (Fig. 1). We used VDJView to integrate immune receptor information with the gene expression profile and available metadata, and performed unsupervised clustering, expanding upon the results depicted in Figure 6a of the original publication [17]. The unsupervised clustering (Supplementary Figure 4) revealed evidence of 8 clusters based on identity (B and T cells), B-cell isotype, tissue of origin and cancer molecular subtype. T cells largely formed a single cluster with marker gene CD96 associated to immune modulation, as well as expression of IL2R-γ and FYB which is known to control IL-2 secretion. The remaining clusters were largely composed of B cells based on tissue of origin, molecular subtype of cancer, and notably a cluster that was composed of IgG1 B cells in metastatic lymph-node of double positive breast cancer, expressing gene signature suggesting they are highly active and differentiated B cells, e.g. plasmablast following a reactivation of memory B cells. In this cluster, the over-expression of PAX5 and TCL1A could also indicate presence of malignant immune cells as these genes are often found in leukemia and likely to contribute to oncogenesis BCL6 [18, 19]. Further analysis of this data is detailed in Supplementary Note 2 (Supplementary Figures 5, 6 and 7).

### Analysis of 10X antigen specific CD8^+^ T cells

To further demonstrate the utility of VDJView, we have analysed the recently published scRNA-seq data with TotalSeq and dextramer stained CD8^+^ T cells. This dataset contains single cell data on over 150,000 CD8^+^ T cells isolated from 4 healthy donors, two of which were CMV positive, 44 dextramers were simultaneously used in each subject to isolate antigen specific T cells across viral infections (CMV (Cytomegalovirus), EBV (Epstein-Barr virus), HPV (Human papillomavirus), Influenza, HIV (Human immunodeficiency virus)), and cancer (e.g., MART, MAGE NY-ESO). We used this data to study the clonal distribution within and across specific antigens and link this information to the gene expression and other metadata.

In this analysis, we uploaded and analysed the TCR sequences and the gene expression matrices available on the 10X Genomics website (https://support.10xgenomics.com/single-cell-vdj/datasets). Utilising the available csv template in VDJView, we generated a third file containing the available metadata for each cell, e.g., subject ID, TotalSeq 15 surface markers including T cell differentiation markers (CD45RA, CD45RO, CCR7) and exhaustion and activation markers such as HLA-DR and PD-1, and tetramers read-counts (HLA-I restricted epitopes), MHC allele and other information. Given the large number of cells in the dataset and the high dimensionality of the transcriptomics data, which can be a limitation for the standard computational resources available to the user, we used VDJView to randomly sample 15,000 cells from each of donor 1, 2 and 3. This allowed us to perform the following analyses on a standard machine with 16GB RAM. For the 15,000 cells from donor 1, we performed quality control on the data, filtering out cells with > 15% mitochondrial genes or abnormally high total expression counts, leaving 11,675 cells. After removing these obvious outliers, contaminants and poor quality cells, we filtered out cells with low tetramer read counts, or tetramer read counts that were not significantly higher than the negative control tetramers (also available in the dataset). This filtering resulted in 3815 antigen specific T cells. Further details on the analysis of data from donor 2 and 3 are provided in Supplementary Note 3.

**Fig. 2:**
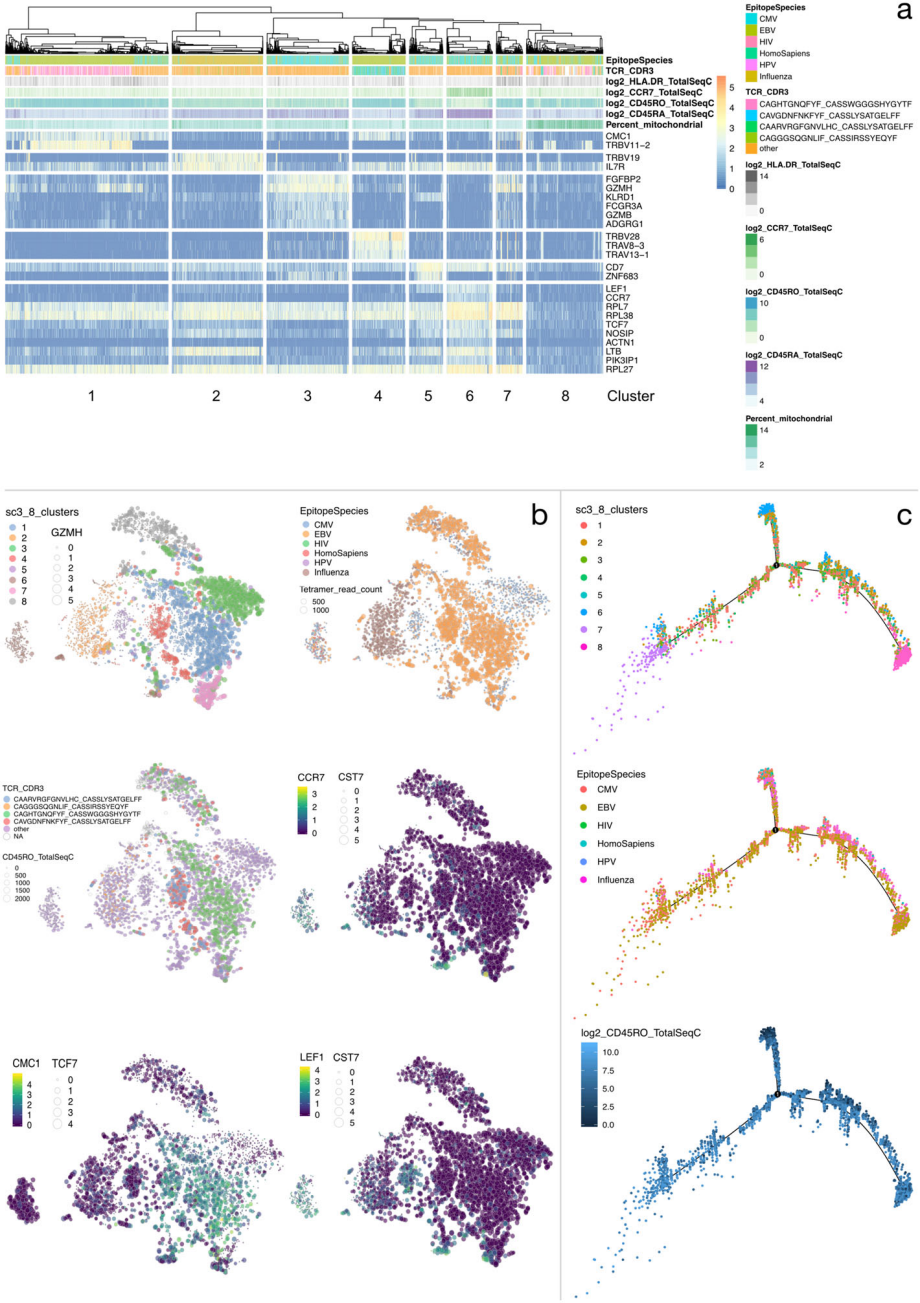
Analysis of CD8^+^ antigen-specific T cells sampled from Donor 1. **a** Unsupervised clustering with k = 8 clusters, *p*-value = 0.01, AUROC = 0.8. Epitope species specificity, the four largest TCR clones, surface protein expression levels, and the percentage of mitochondrial genes are annotated. **b** t-SNE coloured by the results of clustering, epitope species, TCR clone and genes of interest (CCR7, CMC1, LEF1), with point size corresponding to highest tetramer read count of each cell, CD45RO TotalSeq expression, and genes of interest (GZMH, CST7, TCF7), show that clustering is preserved, and that clonally expanded T cells dominate the major clusters. Genes of interest reveal further sub-clusters of cells. **c** Pseudo-time plots reveal a naïve to effector phenotype transition, with cluster preservation at the extremes of each state and a clear trajectory for influenza specific T cells

We used this set to explore the distribution of genes, markers for T cell differentiation, receptor clonotype, and tetramer specificity. Unsupervised analysis (Fig. 2a) revealed 8 clusters with marker genes identifying signatures of cytotoxic activities of CMV, EBV and Influenza specific CD8^+^ T cells, and the presence of memory and naïve T cells (e.g., CCR7^+^ CD45RO^+^ and CCR7^+^ CD45RA^+^), thus, revealing clustering based on epitope specificity, T-cell differentiation and TCR specificity. Specifically, clusters 1 and 4 showed clonally expanded populations of EBV specific memory cells identified by marker genes being TCR V genes and by complementarity-determining region 3 (CDR3) specificity. Interestingly, two similar clusters (3 and 6) of clonally expanded EBV specific memory T cells were observed in the cells isolated from donor 2 (Supplementary Figure 8). These clusters were also marked by TCR V genes and CMC1. Cluster 2 revealed influenza specific memory cells, expressing TRBV19, known to code for a public TCR specific to the highly-conserved M158–66 immunodominant epitope [20]. A similar cluster (cluster 2 in Supplementary Figure 8) was also observed in donor 2, again supporting the homogeneity of immune response again influenza across individuals. Clusters 3, 5, and 6 mostly revealed CMV-specific cells displaying no obvious clonality. These three CMV-specific clusters revealed heterogeneous expression of Granzyme H and B genes, and of transcription factors LEF1, TCF7, and ZNF683 (Hobit), which are regulators of T-cell differentiation. Conversely, when analyzing cells from donor 3 (known to be seropositive for CMV), a large expansion of active (CCL5^+^ NKG7^+^ GZMA^+^ CD45RO^+^ CD45RA^−^**)** CMV-specific cells was observed in clusters 2–5 (Supplementary Figure 9). Evidence of clonal expansion was also observed in clusters 2 and 5 (Supplementary Figure 9). Unsupervised clustering on the integrated data from donors 1 and 3 (Supplementary Figure 10) confirms that the CMV-specific T cells cluster according to donor, despite some similarity in gene signature (JUN^+^ LEF1^+^). The cells in cluster 6 are clearly naïve (CD45RO^−^ CD45RA^+^ CCR7^+^) and consistent with those observed in donor 3 (cluster 1, Supplementary Figure 9). Finally, cluster 7 formed CMV and EBV specific and clonally expanded memory T cells, revealed by the same TCR CDR3 sequence. Notably, despite the filtering of low quality cells, cluster 8 revealed cells with reduced expression of all marker genes, including housekeeping genes RPL7 and RPL27, and with the highest percentage of mitochondrial genes, thus reinforcing the importance of quality control steps in scRNA-seq analysis.

We then utilised the dimensionality reduction features of VDJView to further explore clonality within these subsets. We used the t-SNE plots (Fig. 2b) generated utilising the gene expression profiles to explore protein and tetramer expression, as well as other metadata information. As expected, the clusters identified via SC3 largely formed distinct clusters, with EBV and influenza specific T cells revealing the highest tetramer read counts, suggesting a high binding affinity of these cells for the cognate antigens. Within the CMV and EBV specific T cells, clonally expanded T cells formed larger clusters, suggesting a common gene signature in clonally expanded populations. By marking the expression of genes such as GZMH, LEF1, TCF7, CMC1 and CCR7 gene expression, the t-SNE plots revealed sub-clusters based on the differentiation status of T cells. Finally, we performed pseudo-time analysis (Fig. 2c) to reveal a naïve to effector phenotype transition, shown by the increase in CD45RO expression, which is inversely mirrored in CD45RA expression. This analysis showed that naïve T cells identified in cluster 6 in the SC3 analysis formed a separate branch, while memory T cells were distributed across the pseudo-time structure.

**Fig. 3:**
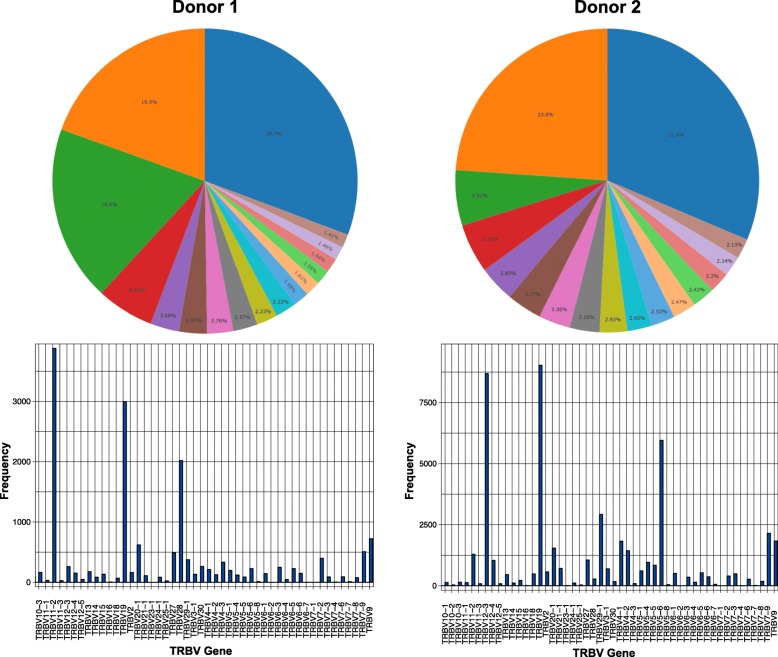
Summary of donor 1 and donor 2 clonal repertoires. Top 16 clones for each donor displayed in pie charts and the TRBV gene usage across all TCR in each donor is detailed in the histograms

We also analysed the TCRs of all T cells from donors 1 and 2. After performing the same quality control and filtering as outlined above, we were left with 55,922 antigen specific T cells (14,199 from donor 1 and 41,723 from donor 2). Both donors displayed clonally expanded populations (Fig. 3), with 3 unique TCR expanded across at least 1000 cells, and over 16 expanded across at least 100 cells. Both donors displayed VDJ gene usage bias, with a relatively high usage of TRBV19 common to both donors. We identified a total of 15,600 unique TCRs, with 411 TCRs common in both donors (Table 2 shows 15 of these). We also found evidence of cross reactive TCR that target different antigens within the same species, or across species, opening further avenues of study.

**Table 2:** TCR clones shared between donor 1 and donor 2, and the species they target with the number of occurrences in each donor

TCR_CDR3	Species	d1	d2
CAGHTGNQFYF_CASSWGGGSHYGYTF	EBV	2379	37
CAVGDNFNKFYF_CASSLYSATGELFF	EBV	1511	23
CAARVRGFGNVLHC_CASSLYSATGELFF	EBV	1442	19
CAASGYDYKLSF_CSVSASGGDEQYF	CMV, EBV	214	10
CAVFLYGNNRLAF_CSVSASGGDEQYF	CMV, EBV	199	11
CAASETSYDKVIF_CASSFSGNTGELFF	EBV	38	5810
CADSGGGADGLTF_CASSLRDGSEAFF	EBV	34	4428
CAASETSYDKVIF_CASSWGGGSHYGYTF	EBV	14	35
CAGAGSQGNLIF_CASSIRSSYEQYF	Influenza	16	345
CAVTDGGSQGNLIF_CASSIRSSYEQYF	Influenza	120	39
CAGAHGSSNTGKLIF_CASSIRSAYEQYF	Influenza	71	48
CAVSGSQGNLIF_CASSIRSSYEQYF	Influenza	10	79
CAAGGSQGNLIF_CASSIRSSYEQYF	Influenza	10	77
CAGGGSQGNLIF_CASSIRSSYEQYF	Influenza	469	1094
CAGGGSQGNLIF_CASSVRSSYEQYF	Influenza	119	72

## Discussion

We have shown that integrating immune receptor and gene expression data with clinical information is useful to discover novel, biologically relevant findings from published data that do not emerge through previous analyses, and to further understand and discover medically relevant mechanisms. VDJView, a unique platform to conduct such analysis, forms an integrated set of known and novel tools that have a flexible design, expanding other tools and providing a robust quantitative framework to generate and study multi-omic immune cell data at the single cell level. VDJView accepts data from numerous different scRNA-seq pipelines, and outputs data that can be extracted in various formats (pdf, csv, R data objects) and used with other software to perform additional analyses. The proposed framework can be utilised by bioinformatics experts to develop and integrate new tools, as well as by clinical scientists and immunologists without profound knowledge of bioinformatics tools. Additionally, we propose that the software is a useful tool for lab-meetings as it promotes an on-the-go type of analysis that is suitable for quick hypothesis testing.

### Limitations

VDJView is developed in R, and therefore it is relatively simple to maintain and install. However, updates to the packages that VDJView utilises may cause dependency issues or loss of function due to code deprecation. This is an issue that requires periodic updates, and while we will maintain the software, we recommend using the suggested R versions. While the software is designed to be intuitive, some statistical and domain knowledge is required to tune parameters such as *p*-values and AUROC in clustering, or perplexity in tSNE, to avoid over-interpretation. The default values of the clustering parameters are chosen conservatively to prevent data over-fitting, and the default tSNE perplexity scales up with data size to prevent the observation of small clot-like structures. Additionally, VDJView does not perform any batch correction. As such, any technical variation in the data should be corrected for prior to uploading. Given the significant technical noise that characterises scRNA-seq data, users are advised to consult statistical experts. VDJView will be maintained monthly and new tools will be integrated according to the development of software packages in the field, and the feedback received from users of the software.

## Conclusions

VDJView is a complete software package for downstream analysis of single cell gene expression, immune receptor and metadata, which allows exploratory and hypothesis driven analysis of multi-omic datasets. In summary, VDJView has the potential to allow clinical and experimental researchers to utilise complex genomics data to test biologically relevant questions.

## Availability and requirements

**Project name:** VDJView

**Project home page:** https://bitbucket.org/kirbyvisp/vdjview

**Operating system(s):** Linux, MacOS, with major features functional on Windows

**Programming language:** R

**Other requirements:** R 3.5.2 or higher

**License:** GNU

**Any restrictions to use by non-academics:** None

## Supplementary information

**Additional file 1.** Supplementary Material.

## Data Availability

All data and metadata presented are publicly available and have been compiled into the following repository for ease of access: https://drive.google.com/drive/folders/1ATRZ239ubNu8Jv7Zy71WFylmGdC0TALt

## Abbreviations

AUROC: Area under the receiver operating characteristic
BCR: B cell receptor
CDR3: Complementarity-determining region 3
CMV: Cytomegalovirus
EBV: Epstein-Barr virus
HIV: Human immunodeficiency virus
HPV: Human papillomavirus
PCA: Principle component analysis
scRNA-seq: Single cell RNA-sequencing
TCR: T cell receptor
tSNE: T-distributed stochastic neighbour embedding
UMAP: Uniform manifold approximation and projection
